# Klotho as Potential Autophagy Regulator and Therapeutic Target

**DOI:** 10.3389/fphar.2021.755366

**Published:** 2021-10-19

**Authors:** Hongjing Zhou, Shiyun Pu, Houfeng Zhou, Yuanxin Guo

**Affiliations:** Department of Pharmacy, Chengdu Fifth People’s Hospital, Chengdu University of Traditional Chinese Medicine, Chengdu, China

**Keywords:** klotho, autophagy, alzhaimer’s disease, kidney injury, cancer, chronic obstructive pulmonary disease, vascular disease, muscular dystrophy

## Abstract

The protein Klotho can significantly delay aging, so it has attracted widespread attention. Abnormal downregulation of Klotho has been detected in several aging-related diseases, such as Alzheimer’s disease, kidney injury, cancer, chronic obstructive pulmonary disease (COPD), vascular disease, muscular dystrophy and diabetes. Conversely, many exogenous and endogenous factors, several drugs, lifestyle changes and genetic manipulations were reported to exert therapeutic effects through increasing Klotho expression. In recent years, Klotho has been identified as a potential autophagy regulator. How Klotho may contribute to reversing the effects of aging and disease became clearer when it was linked to autophagy, the process in which eukaryotic cells clear away dysfunctional proteins and damaged organelles: the abovementioned diseases involve abnormal autophagy. Interestingly, growing evidence indicates that Klotho plays a dual role as inducer or inhibitor of autophagy in different physiological or pathological conditions through its influence on IGF-1/PI3K/Akt/mTOR signaling pathway, Beclin 1 expression and activity, as well as aldosterone level, which can help restore autophagy to beneficial levels. The present review examines the role of Klotho in regulating autophagy in Alzheimer’s disease, kidney injury, cancer, COPD, vascular disease, muscular dystrophy and diabetes. Targeting Klotho may provide a new perspective for preventing and treating aging-related diseases.

## Introduction

In 1997, Kuro-o and colleagues discovered a gene whose deletion shortened the mouse lifespan to 8–9 weeks and led to multiple complications of premature aging, such as gonadal dysplasia, skin atrophy, osteoporosis, atherosclerosis, hypoglycemia, and emphysema (Kuro-o et al., 1997). Overexpressing the gene in mice significantly extended their lifespan (Kurosu et al., 2005). The researchers named the gene and its encoded protein “Klotho”, and some later studies referred to it as α-Klotho, after the isolation of other Klotho proteins, including β-Klotho (Ito et al., 2000), KLPH (Ito et al., 2002) and Klotho-related protein (Klrp) (Hayashi and Ito 2016). α-Klotho and β-Klotho have high homology, but their distribution and functions are very different. α-Klotho is expressed abundantly in choroid plexus epithelial cells of the brain and distal convoluted tubules of the kidney, and at low levels in the pituitary, skeletal muscle, pancreas, aorta, testis, ovary, placenta and thyroid gland (Kuro-o et al., 1997; Li et al., 2004; Lim et al., 2015). It participates in Ca^2+^ and phosphate homeostasis, inhibits oxidative damage and inflammation, promotes myelination and long-term enhancement in neurons, and protects stem cells (Liu et al., 2007; Martin et al., 2012; Chen et al., 2013; Xu and Sun 2015; Zhou et al., 2017; Zhou et al., 2018). β-Klotho, in contrast, is expressed mainly in the yolk sac, gut, brown and white adipose tissues, liver and pancreas. It participates in metabolic regulation, glucose uptake, bile acid synthesis and fatty acid metabolism (Ito et al., 2000). Klrp was identified as a cytosolic neutral beta-glucosylceramidase, and it plays a role in glycosphingolipid metabolism and function (Hayashi and Ito 2016). KLPH is a novel mammalian family 1 glycosidase-like protein, expressed predominantly in the kidney and skin (Ito et al., 2002). The present review focuses on α-Klotho, hereafter referred to simply as Klotho.

As an anti-aging protein, Klotho expression decreases with age, and its underexpression has been reported in many aging-related diseases such as Alzheimer’s disease, kidney disease, chronic obstructive pulmonary disease (COPD), certain cancers, cardiovascular and cerebrovascular diseases, as well as diabetes and its complications (Duce et al., 2008; Kuro-o 2012; Semba et al., 2014; Akasaka-Manya et al., 2016; Zhou et al., 2018; Lim et al., 2019). Progression of these diseases and poor outcomes of patients are associated with downregulation of Klotho expression. Conversely, its overexpression can exert therapeutic effects, such as mitigating the deposition of amyloid-β and other pathological changes related to Alzheimer’s disease, delaying progression from acute kidney injury to chronic kidney disease, as well as inhibiting tumor proliferation and drug resistance. Interestingly, recent work has shown a correlation between Klotho expression and changes in autophagy activity in some diseases (Table 1). Studies have linked Klotho’s protective effects to regulation of autophagy: higher expression is associated with improvement of abnormal autophagy, while lower expression is associated with aggravation of abnormal autophagy (Table 2).

**TABLE 1:** Correlation between Klotho expression and autophagy activity in certain diseases, as reported in observational studies.

Diseases	Klotho expression	Changes in autophagy indicators	Autophagy activity
Alzheimer’s disease Zeng et al. (2019)	↓	LC3II/LC3I ↑, p62 ↑, autophagosomes ↑, autolysosomes ↓	dysfunction
Ischemia-reperfusion induced acute kidney injury Shi et al. (2016); Chen et al. (2017); Li et al. (2020)	↓	RFP-LC3 ↑, GFP-LC3 ↑, LC3II ↑, LC3II/LC3I ↑, Beclin 1↑, p62 ↓	↑
Sepsis-induced acute kidney injury Chen et al. (2018a)	↓	LC3II/LC3I ↑, p62 ↓	↑
Head and neck squamous cell carcinoma Zhu et al. (2019)	↑	LC3 ↑	↑
	↓	LC3 ↓	↓
Drug-resistant lung cancer Chen et al. (2016)	↓	Beclin1 ↑, LC3II ↑	↑
Chronic obstructive pulmonary disease Monick et al. (2010)	↓	LC3II/LC3I ↑, p62 ↑, autophagosomes ↑	dysfuction
Type 2 diabetes mellitus Lin and Sun (2015)	↓	LC3 ↓	↓

**TABLE 2:** Regulatory effects of Klotho on autophagy in various diseases, as reported in interventional studies that manipulating Klotho expression.

Organ or tissue	Disease or disease models	Klotho intervention	Intervention strategy	Changes in autophagy indicators	Autophagy activity	Disease outcome
Brain	Alzheimer’s disease Zeng et al. (2019)	↑	Overexpression	LC3II/LC3I ↑, p62 ↓, autolysosome↑	↑	good
	Amyloid-β_1-42_ fibril-treated BV2 cells Zeng et al., 2019	↑	Overexpression and Recombinant Klotho protein	LC3II/LC3I ↑, p62 ↓	↑	good
Kidney	Basic state of Klotho mutant mice and transgenic mice Shi et al. (2016)	↓	Klotho gene mutant	LC3II/LC3 ↓, p62 ↑	↓	—
		↑	Transgenic mice line	LC3II/LC3I ↑, p62 ↓	↑	—
	Ischemia/reperfusion induced acute kidney injury Shi et al. (2016); Chen et al. (2017)	↓	Klotho gene mutant	LC3II/LC3I ↓, p62 ↑, RFP-LC3 ↓, autolysosome ↓, autophagosomes ↓	↓	poor
		↑	Transgenic mouse line	LC3II/LC3I ↑, p62 ↓, RFP-LC3 ↑, Beclin1/Bcl2 complex ↓, autolysosome↑, autophagosomes ↑	↑	good
		↑	Decrease the methylation of Klotho	Beclin1 ↑, LC3 ↑	↑	good
	Collagen I accumulation in opossum kidney cell Shi et al. (2016)	↑	Recombinant Klotho protein	LC3II/LC3I ↑, p62 ↓	↑	good
	Cecal ligation and puncture-induced acute kidney injury (Chen et al., 2018b)	↑	Recombinant Klotho protein	LC3II/LC3I unchanged, p62 unchanged	unchanged	—
	LPS-treated HK-2 cells Chen et al. (2018b)	↑	Recombinant Klotho protein	LC3II/LC3I unchanged, p62 unchanged	unchanged	—
Tumor	Hepatoma Shu et al. (2013)	↑	Overexpression	LC3II ↑, LC3I ↑	↑	good
	Gastric cancer Xie et al. (2013b)	↑	DNA demethylating agent	LC3II/LC3I ↑	↑	good
	Drug-resistant lung cancer cells Chen et al. (2016)	↑	Overexpression	Beclin1 ↓, LC3II ↓	↓	good
Lung	Cigarette smoke extract-treated murine alveolar macrophage cell line Li et al. (2017b)	↑	Recombinant Klotho protein	LC3II/LC3I ↓	↓	NR
		↓	Klotho-siRNA	LC3II/LC3I ↑	↑	NR
Artery	Hypertension (arterial stiffness) Chen et al. (2015); Chen and Sun (2019)	↓	Klotho gene mutant	LC3II ↑, LC3II/LC3I ↑, Beclin 1 ↑, p62 ↓	↑	poor
	Basic state of mouse vascular aortic smooth muscle cells Chen and Sun (2019)	↑	Recombinant secreted Klotho protein	LC3II/LC3I ↓, Beclin 1 ↓, p62 ↑	↓	—
		↓	Klotho-deficient medium	LC3II ↑, Beclin 1↑, p62 ↓	↑	—
Muscle	Muscular dystrophy (masseter and tongue) Iida et al. (2011)	↓	Klotho gene mutant	LC3II/LC3I ↑, p62 ↓, Gabrap ↑	↑	poor
Islet	T2DM in *db/db* mice Lin and Sun (2015)	↑	Overexpression	LC3 ↑	↑	good

NR, not reported.

Autophagy, a programmed process of self-digestion that degrades misfolded and aging proteins, damaged organelles, and other abnormal cell components, is crucial for maintaining cell homeostasis. However, abnormal autophagy, which can be excessive or insufficient, contributes to various diseases, especially those related to aging, including cancer (Akkoc and Gozuacik 2018), cardiovascular disease (Shirakabe et al., 2016), COPD (Racanelli et al., 2018) and neurodegeneration (Yang et al., 2011; Wolfe et al., 2013).

The present review summarizes the state of knowledge about the potential role of Klotho in regulating autophagy in Alzheimer’s disease, kidney injury, cancer, COPD, vascular disease, muscular dystrophy and diabetes. These considerations may lead to strategies for targeting Klotho in aging-related diseases.

### Klotho and Regulation of Its Expression

The Klotho gene is about 50 kb long, and two mRNA transcripts can arise through alternative splicing: one generates the type I transmembrane protein (130 kDa), the other is assumed to generate a secreted protein (70 kDa) (Shiraki-Iida et al., 1998). Although the concept of “secreted Klotho protein” was first proposed in 1998, the existence of this protein remains controversial based on current researches (Masso et al., 2015; Mencke et al., 2017a; Jadhav et al., 2021; Li et al., 2021). Transmembrane Klotho protein is expressed mainly in choroid plexus epithelial cells of the brain and the distal convoluted tubules of the kidney. The extracellular region of transmembrane Klotho protein can be cleaved by α- and β-secretases, and eventually finds its way into blood, urine and cerebrospinal fluid (Chen et al., 2007; Bloch et al., 2009). This cleaved Klotho protein is commonly known as the soluble Klotho.

Klotho expression is influenced by many physiological and pathological conditions. Expression of Klotho in the brain, kidney, heart sinoatrial node, liver and serum decrease substantially with age in animals and humans (Nabeshima 2002; Xiao et al., 2004; Shih and Yen 2007; Duce et al., 2008; Yamazaki et al., 2010; Semba et al., 2014; Akasaka-Manya et al., 2016; Behringer et al., 2018; Zhou et al., 2018). In addition, oxidative stress, inflammation, angiotensin II, aldosterone, and albuminuria suppress Klotho expression (Kanbay et al., 2021). The protein is also underexpressed in many diseases, including Alzheimer’s disease (Kuang et al., 2017; Zeng et al., 2019), acute and chronic kidney diseases (Koh et al., 2001; Zuo et al., 2011; Hu et al., 2013; Kitagawa et al., 2013), COPD (Gao et al., 2015), diabetes (Takenaka et al., 2019; Typiak and Piwkowska 2021), some cancers (Xie et al., 2013a) and a variety of vascular pathologies including arterial stiffness, atherosclerosis and stroke (Lim et al., 2019; Memmos et al., 2019).

Interestingly, increasing or restoring expression of Klotho slows down aging and mitigates the pathology of the abovementioned diseases, making Klotho a potential therapeutic target. Indeed, numerous strategies for upregulating or restoring Klotho expression have been reported. Many exogenous and endogenous factors have been shown to upregulate it, including ligustilide, oleanolic acid, acetyl-11-keto-β-boswelic acid, alginate oligosaccharide, baicalin, daidzein, curcumin, Necrostatin-1, ginsenoside-Rg1, salvianolic acid A, ursolic acid, rhein, the circular RNA “ITCH”, and vitamin D (Kuang et al., 2017; Long et al., 2018; He et al., 2018; Liu et al., 2018; Pan et al., 2021; Zhang et al., 2020; Zivanovic et al., 2019; Mansoor et al., 2018; Ning et al., 2018; Li et al., 2018; Zhang S. et al., 2018; Gharibi et al., 2018; Zhang et al., 2016). Several drugs also upregulate Klotho, such as infliximab (Younis et al., 2021), pioglitazone (Maquigussa et al., 2018; Shen et al., 2018), empagliflozin (Abbas et al., 2018), sulodexide (Liu et al., 2017), and simvastatin (Adeli et al., 2017). Lifestyle changes such as aerobic exercise (Ji et al., 2018) and intermittent fasting (Dias et al., 2021), can also increase Klotho expression. Various genetic approaches can be used to express the protein in tissues, including CRISPR and recombinant adeno- and lentiviruses (Chen X. et al., 2018; Masso et al., 2018; Zhao et al., 2020).

### Autophagy

Recent studies have suggested that Klotho may regulate autophagy in various tissues. This review focuses on Klotho’s role in macroautophagy, which delivers degradation substrates to lysosomes, forming an intermediate structure called the autophagosome. Macroautophagy occurs in three steps: 1) encapsulation of abnormal proteins and damaged organelles into autophagosomes, 2) fusion of autophagosomes with lysosomes to form autolysosomes, and 3) degradation of the contents within autolysosomes (Parzych and Klionsky 2014).

Several autophagy proteins, such as LC3 and SQSTM1/p62, are commonly used as markers to track the process. LC3 plays a key role in autophagosome maturation. Precursor forms of LC3 are specifically cleaved by ATG4 family proteins to form LC3-I, which has an exposed carboxyl terminal glycine that is conjugated to phosphatidylethanolamine to form LC3-II. LC3-II is bound tightly to both the inner and outer surfaces of the autophagosomal membrane, and it participates in autophagosome formation (Kabeya et al., 2000). Thus, an increase in the conversion of LC3-I to LC3-II is generally considered to reflect activation of autophagy. However, an accumulation of LC3-II can also occur when downstream steps are blocked, reflecting ineffective autophagy. p62 is one of the autophagy-specific substrates, so its level negatively correlate with the activity of autophagy (Bjorkoy et al., 2005; Pankiv et al., 2007).

The body regulates autophagy primarily through signaling via type I phosphoinositide 3-kinase (PI3K) and Akt. Akt phosphorylates mTOR, a serine/threonine protein kinase that is highly conserved in eukaryotic cells, which in turn inhibits autophagy (Jung et al., 2009; Jung et al., 2010). This signaling pathway can be induced by IGF-1 (Troncoso et al., 2013). Conversely, inhibition of PI3K/Akt signaling inhibits the phosphorylation of mTOR, thereby enhancing autophagy (Wang et al., 2015). Besides, autophagy can also be induced by RAS/RAF/MEK/ERK signaling pathway (Zhang et al., 2017; Sooro et al., 2018) and aldosterone (Yang et al., 2016; Luo et al., 2017).

### Klotho and Autophagy

#### Klotho and Autophagy in Alzheimer’s Disease

Autophagy is the main way for the central nervous system to clear away abnormal proteins such as amyloid-β and damaged organelles (Wolfe et al., 2013; Plaza-Zabala et al., 2017). Autophagy is impaired in the brains of patients and animal models with Alzheimer’s disease (Yang et al., 2011; Castellazzi et al., 2019; Pomilio et al., 2020), and this defect is associated with low Klotho expression and may be related to amyloid-β deposition (Zeng et al., 2019). The APP^swe^/PS1^dE9^ transgenic mouse (hereafter referred to as the “APP/PS1 mouse”) harbors mutant mouse/human APP (Swedish K595N/M596L) and PS1 genes (PS1-dE9) and is commonly used as an animal model of Alzheimer’s disease. These mice show abundant abnormal deposition of amyloid-β in the brain. They also show lower Klotho expression and greater autophagy in the brain than wild-type animals of the same age (Zeng et al., 2019). The increased autophagy seems to be ineffective, as reflected in the simultaneous increase in the LC3-II/LC3-I ratio, the number of autophagosomes, and the level of p62 (Zeng et al., 2019).

Upregulating Klotho can rescue “healthy” autophagy, reflected in an increase in the LC3-II/LC3-I ratio and number of autolysosomes with concomitant decrease in p62 levels (Zeng et al., 2019). These changes are associated with milder Alzheimer’s neuropathology and less amyloid-β deposition. These changes also involve inhibition of PI3K/Akt/mTOR signaling (Zeng et al., 2019), suggesting that Klotho restores normal autophagy in the central nervous system by regulating PI3K/Akt/mTOR signaling, which helps clear away amyloid-β.

A recombinant form of mouse Klotho protein containing the ectodomain promotes phagocytosis and the subsequent lysosomal degradation of amyloid-β_1-42_ fibrils (fAβ) in cultures of BV2 mouse microglia (Zeng et al., 2019). Overexpression of Klotho in fAβ-treated BV2 cells induces substantial autophagy, as reflected in an elevated LC3-II/LC3-I ratio and reduced p62 levels, through a mechanism that may involve inhibition of Akt/mTOR (Zeng et al., 2019).

The other important pathological change typical of Alzheimer’s disease is intracellular neurofibrillary tangles (NFTs), which are induced by hyperphosphorylation of the tau protein (Gao et al., 2018). Hyperphosphosyrlated tau is removed in part through autophagy (Xin et al., 2018; Bao et al., 2020), and levels of this protein reduced by overexpressing Klotho (Zeng et al., 2019).

Lipofuscin, an electron-dense substance that is thought to consist of oxidized proteins and lipids, is deposited in senescent cells and cannot be further degraded by lysosomes (Terman and Brunk 1998; Double et al., 2008). The accumulation of lipofuscin in the central nervous system is associated with neuronal loss, glial proliferation and activation (Moreno-Garcia et al., 2018). Macroautophagy may participate in the formation of lipofuscin or may be responsible for the uptake of lipofuscin into lysosomes (Hohn and Grune 2013). APP/PS1 mice show abnormal accumulation of lipofuscin in the brain, which overexpression of Klotho alleviates (Zeng et al., 2019).

Abnormal autophagy seems to be involved in the occurrence and development of a variety of neuropathological changes including deposition of amyloid-β, formation of NFTs and abnormal accumulation of lipofuscin. Klotho expression seems to mitigate these processes by restoring or increasing autophagy activity. At the same time, Klotho overexpression may also increase the clearance of amyloid-β by affecting the expression of amyloid-β transporters including LRP1, P-gp, ABCA1 and RAGE (Zhao et al., 2020). Therefore, more studies are needed to explore how precisely Klotho may alleviate AD pathology.

#### Klotho and Autophagy in Kidney Injury

Klotho expression in the kidney decreases not only during aging (Manya et al., 2010) but also in acute kidney injury (Panesso et al., 2014) and chronic kidney disease (Hu et al., 2011). One study showed that giving hydrogen-rich saline to a mouse model of acute kidney injury upregulated Klotho expression and protected the kidney from further damage (Chen et al., 2017). These changes were associated with increases in LC3 and Beclin1, implying an increase of autophagy in the kidneys, though that study did not explore this possibility further (Chen et al., 2017).

Consistent with the idea that Klotho helps drive autophagy, another study showed that autophagy can be induced by Klotho at the baseline unperturbed state in the kidney of *Tg-Kl* (transgenic mice expressing 150% the normal level of Klotho), while inhibited by Klotho deficiency in the kidney of *kl/+* (Klotho gene mutant mice expressing 50% the normal level of Klotho) (Shi et al., 2016). Further study showed that acute kidney injury in mice activated autophagy in the kidneys, and that this activation was greater in *Tg-Kl* (Shi et al., 2016). The greater activation of autophagy induced by Klotho was associated with greater mitigation of ischemia/reperfusion-induced acute kidney injury, and may delay progression from acute kidney injury to chronic kidney disease through clearance of type I collagen (Shi et al., 2016).

These findings in animals were corroborated and extended in culture studies. Adding Klotho to the culture medium of a proximal tubular cell line from opossum kidney increased the LC3-II/LC3-I ratio and the number of autophagosomes while reducing the p62 level, indicating higher baseline autophagic flux (Shi et al., 2016). This Klotho-induced elevation of autophagy flux was blunted by bafilomycin A1, an autophagy inhibitor that inhibits the fusion of autophagosomes and lysosomes (Shi et al., 2016). In addition, bafilomycin A1 and 3-methyladenine, which inhibit the formation of autophagosomes, blunted the protective effect of Klotho in hydrogen peroxide-induced injury and reduced the accumulation of collagen I by reducing autophagy activity (Shi et al., 2016).

These results suggest that insufficient Klotho expression can lead to inadequate autophagy in the kidney, which Klotho upregulation can improve to a certain extent. These effects may involve Beclin 1-dependent renal protection by Klotho. Beclin1, which is negatively regulated by binging to Bcl2, acts as a central regulator of autophagy in mammalian cells (Qu et al., 2003; Pattingre et al., 2005). Disruption of the Beclin 1/Bcl-2 autophagy regulatory complex promotes longevity in mice (Fernandez et al., 2018). *Tg-Kl* mice showed a decrease in Beclin 1/Bcl2 complex in the kidney (Li et al., 2020). A recombinant form of mouse Klotho containing the ectodomain also downregulated the Beclin 1/Bcl2 complex in the kidney (Li et al., 2020). Surprisingly, low Beclin1 activity in mice was associated with weaker ability of exogenous Klotho protein or high Klotho expression to protect kidneys against ischemia-reperfusion injury, suggesting that Beclin1 may help mediate Klotho’s autophagy-dependent effects. (Li et al., 2020). These effects of Klotho may also involve the IGF-1R/Akt/mTOR signaling pathway, since siRNA-mediated knockdown of Klotho significantly activated such signaling in HEK293T cells (Kuang et al., 2017).

These findings suggest that Klotho protects kidneys from disease in part by enhancing autophagy activity. However, not all of the protective effects of Klotho in kidney involve regulation of autophagy: one study showed that Klotho can mitigate sepsis-induced acute kidney injury without affecting levels of autophagy in the kidney. Therefore, more studies are needed to explore how Klotho regulates autophagy as well as potentially other processes in the kidney.

#### Klotho and Autophagy in Cancer

Abnormal autophagy has been detected in various types of tumors, and the dysregulation of this process may promote tumor occurrence and development, as well as the emergence of drug resistance (Rebecca and Amaravadi 2016; Li L. et al., 2017; Kimmelman and White 2017).

Studies in various types of cancer suggest that Klotho acts as a tumor suppressor (Xie et al., 2013a). The abnormal autophagy in tumors may be associated with Klotho underexpression, which has been documented in hepatocellular carcinoma (Shu et al., 2013), head and neck squamous cell carcinoma (HNSCC) (Zhu et al., 2019), gastric cancer (Xie et al., 2013b), as well as lung cancer (Chen et al., 2016). For example, in cultures of the human hepatoma cell lines HepG2 and MHCC-97-H, restoration of Klotho significantly inhibited their cell proliferation (Shu et al., 2013). Such restoration also increased levels of LC3-II and LC3-I, which was reversed by autophagy inhibitors (Shu et al., 2013). As another example, Klotho levels correlate positively with levels of LC3 in patients with HNSCC, and low Klotho expression may predict worse prognosis in that disease (Zhu et al., 2019).

Klotho downregulation in cancer seems to be the result of promoter methylation and histone modification (Pan et al., 2011; Wang et al., 2011; Rubinek et al., 2012). There is an obvious negative correlation between Klotho expression and its DNA methylation in HNSCC, suggesting that Klotho DNA methylation leads to silencing of its expression (Zhu et al., 2019). High Klotho gene methylation is negatively associated with LC3 expression, making it a potential biomarker for worse prognosis in HNSCC (Zhu et al., 2019). Treating gastric cancer cells with the demethylating reagent 5-Aza restored Klotho expression, increasing the ratio of LC3-II/LC3-I, indicating activation of autophagy (Xie et al., 2013b). These effects of 5-Aza were partially reversed by the autophagy inhibitor 3-methyladenine (Xie et al., 2013b).

Based on these studies, the link between Klotho upregulation and activation of autophagy appears to depend on the downregulation of the IGF-1R/PI3K/Akt/mTOR signaling pathway (Xie et al., 2013b; Shu et al., 2013). However, the downregulation of the ERK signaling pathway have also been detected in Klotho-induced autophagy (Shu et al., 2013). This effect is inconsistent with the later studies: autophagy can be induced by RAS/RAF/MEK/ERK signaling pathway (Zhang et al., 2017; Sooro et al., 2018). The regulation of Klotho on ERK signaling pathway does not seem to affect the results of Klotho on autophagy, suggesting that multiple signaling pathways exist, and which signaling pathway is predominate may depend on the favorable outcome. Therefore, the molecular mechanisms of Klotho-induced autophagy in cancer need to be further researched.

By regulating autophagy, Klotho may also influence the emergence of cancer drug resistance (Chen et al., 2016). In lung cancer, drug-resistant tumor cells express significantly less Klotho than drug-sensitive lines and show greater autophagy, reflected in upregulation of Beclin1 and LC3-II (Chen et al., 2016). Overexpressing Klotho in drug-resistant cells inhibited autophagy to a similar extent as 3-methyladenine, partially restoring drug sensitivity (Chen et al., 2016).

These findings suggest that restoration of Klotho expresssion suppresses tumor growth by increasing autophagy activity. On the other hand, the ability of Klotho to restore drug sensitivity appears to involve downregulation of autophagy activity in some cases, highlighting the dual role of Klotho in regulating autophagy in tumor cells. Autophagy is a “double-edged sword” for tumors, so depending on the circumstances, stimulating or inhibiting it may be an effective therapy. The choice of whether to stimulate or inhibit autophagy is important: some autophagy modulators, such as chloroquine, can trigger serious autophagy-related side effects when used as anticancer drugs (Kimura et al., 2013). Thus, the dual role of Klotho in regulating autophagy, which can restore autophagy to beneficial levels, makes it a highly attractive target in anti-tumor therapy, not to mention that Klotho can also exert inhibitory effects on tumors through other biological activity, such as inhibition of Wnt and TGF-β1 signaling pathways (Rubinek and Wolf 2016).

#### Klotho and Autophagy in Chronic Obstructive Pulmonary Disease

COPD is one of the most frequent causes of morbidity and mortality in the world, and one of the major risk factors for the disease is exposure to cigarette smoke (Eisner et al., 2010; van Koeverden et al., 2015). Such exposure has been linked to Klotho underexpression, which has been reported in lung macrophages of smokers with or without COPD, in mouse alveolar macrophages, as well as in bronchial epithelial cells from individuals with COPD (Li et al., 2015; Krick et al., 2018). Klotho overexpression decreased sensitivity to cigarette smoke-induced cell death *in vitro* (Blake et al., 2015). At the same time, such exposure to cigarette smoke appears to activate autophagy (Li YJ. et al., 2017). For example, exposing primary cultures of human bronchial epithelial cells to cigarette smoke extract transiently activated autophagy, leading to cell senescence (Fujii et al., 2012). Exposing mouse alveolar macrophages to cigarette smoke extract significantly increased the LC3-II/LC3-I ratio (Monick et al., 2010). The resulting autophagy appears to be abnormal: alveolar macrophages from smokers show autophagosome and p62 accumulation due to blocked fusion of autophagosomes and lysosomes, as well as decreased clearance of long lived proteins (Monick et al., 2010). These studies link the pathogenesis of COPD to abnormal autophagy due to downregulation of Klotho.

Consistent with this idea, pretreating mouse alveolar macrophages with recombinant Klotho blocked the exposure-induced increase in LC3-II/LC3-I ratio, while pretreating them with siRNA to knock down Klotho exacerbated the exposure-induced increase (Li YJ. et al., 2017). Researchers attributed these effects to inhibition of IGF-1 and its downstream Akt and ERK phosphorylation (Li YJ. et al., 2017), which was inconsistent with other studies: autophagy can be negatively regulated by IGF-1 signaling pathway (Jia et al., 2006; Bitto et al., 2010; Troncoso et al., 2012). The possible reason for this is that other molecular mechanisms exist. Besides, those studies measured only LC3-II/LC3-I ratio, which is not enough to assess autophagy activity. These studies identify Klotho as a therapeutic target for inhibiting abnormal activation of autophagy in lung disease. However, more studies are needed to verify the effects of Klotho by measuring autophagy flux and to explore the underlying molecular mechanisms.

Despite the better clinical condition associated with higher Klotho expression, plasma levels of the protein may not be useful as a biomarker for stable COPD because the levels do not vary during rehabilitation, nor do they correlate with clinical parameters (Pako et al., 2017). Thus, future studies may wish to focus more on the role of Klotho in lung tissue, such as lung macrophages, alveolar macrophages and bronchial epithelial cells.

#### Klotho and Autophagy in Vascular Disease

Vascular aging and dysfunction are key characteristics of cardiovascular and cerebrovascular diseases such as hypertension, atherosclerosis and stroke (Gimbrone and Garcia-Cardena 2016; Hu et al., 2017; Petrie et al., 2018). Abnormal autophagy may impair vessel wall function and initiate or aggravate vascular diseases. Interestingly, Klotho deficiency is associated with medial calcification, intima hyperplasia, endothelial dysfunction, arterial stiffening, hypertension, and impaired vasculogenesis (Mencke et al., 2017b). Thus, researchers have begun to explore the association between Klotho and autophagy in vascular diseases.

Arterial stiffness, one of the earliest detectable manifestations of adverse structural and functional changes within the vessel wall, was reported to be a major risk factor for hypertension, stroke and ischemic heart disease (Safar 2001; Cavalcante et al., 2011; Sun 2015). Enhanced autophagic activity contributes to arterial stiffening by altering the activity of MMP-9 as well as expression of TGF-β1 and the transcription factors RUNX2 and scleraxis, ultimately inducing elastin degradation and increasing the accumulation of collagen (Chen et al., 2015; Chen and Sun 2019; Kanbay et al., 2021).

Serum levels of Klotho are significantly decreased in patients with arterial stiffness and hypertension (Kitagawa et al., 2013), and Klotho deficiency has been shown to induce autophagy, which injures vasculature and causes arterial stiffening and hypertension (Chen et al., 2015; Chen and Sun 2019). In cultures of continuous mouse vascular aortic smooth muscle cells, recombinant secreted Klotho protein decreased LC3-II expression and increased p62 expression, suggesting inhibited autophagy. Conversely, Klotho-deficient medium increased LC3-II expression and decreased p62 expression. (Chen and Sun 2019). Klotho ability to regulate autophagy in such cells may involve Beclin1 (Chen and Sun 2019). In mice heterozygous for mutant Klotho [KL (+/-)], autophagy activation was enhanced, as evidenced by increased expression of LC3-II and decreased p62 level in the aorta. This was associated with arterial remodeling: upregulation of collagen I, downregulation of elastin and a decrease in the ratio of elastin to collagen (Chen and Sun 2019). These effects of Klotho deficiency were abolished by the autophagy inhibitors chloroquine, which blocks the last step in autophagy and thereby leads to the accumulation of ineffective autophagosomes; and eplerenone, which blocks aldosterone receptors. (Chen et al., 2015; Chen and Sun 2019). Aldosterone may induce autophagy to cause an aggravation of diseases (Yang et al., 2016; Luo et al., 2017), and KL (+/-) mice showed elevated serum levels of aldosterone. These results suggest that Klotho deficiency may decrease elastin levels in smooth muscle cells by upregulating aldosterone and thereby inducing autophagy (Chen et al., 2015). Other mediators likely also exist, since the changes associated with Klotho deficiency were blocked by the specific SIRT1 activator SRT1720l (Gao et al., 2016). These findings suggest that exogenous Klotho inhibits autophagy in aorta, while Klotho deficiency induces it, but the underlying molecular mechanisms need to be further studied.

Atherosclerosis is one of the main mechanisms of cardiovascular disease. Basal autophagy is atheroprotective during early atherosclerosis, but it becomes dysfunctional in advanced atherosclerotic plaques (De Meyer et al., 2015). Circulating Klotho levels and Klotho expression in peripheral mononuclear blood cells were significantly lower in individuals with atherosclerosis than in those without it (Yu et al., 2018; Kazemi Fard et al., 2021). It would be interesting to further explore whether Klotho plays a role in dysfunctional autophagy in Atherosclerosis.

Accumulating evidence shows that autophagy is activated in brain microvascular cells, following ischemic stroke (Zhang Z. et al., 2018; Yang et al., 2020). Besides, autophagy alleviates hypoxia-induced blood-brain barrier injury (Yang et al., 2020). Interestingly, in patients and animal models of acute ischemic stroke, higher Klotho levels are associated with good functional outcome, while lower Klotho levels are associated with poor outcome (Zhou et al., 2017; Lee et al., 2019). Further studies are needed to explore potential associations between Klotho and autophagy in stroke.

#### Klotho and Autophagy in Muscular Dystrophy

Klotho plays an important role in maintaining normal muscle function. Klotho-deficient mice show a marked decline in muscle strength and running endurance, as well as severely impaired regeneration of skeletal muscle (Phelps et al., 2013; Ahrens et al., 2018). Klotho gene silencing promoted pathology in the mdx mouse model of Duchenne muscular dystrophy (Wehling-Henricks et al., 2016). Conversely, recombinant Klotho protein stimulated muscle regeneration in the animals, partly by rejuvenating aged muscle stem cells (Ahrens et al., 2018). In fact, skeletal muscle activity may modulate Klotho expression: acute exercise sessions increased levels of circulating Klotho in young and aged mice as well as humans (Avin et al., 2014). These findings make Klotho a potential target for the prevention and treatment of skeletal muscle-related diseases.

Autophagy is necessary to maintain normal muscle function: excessive autophagy leads to loss of muscle mass (Sandri 2010). However, little is known about how Klotho regulates autophagy in muscle tissue. In mice homozygous for a mutated form of the Klotho gene that substantially shortened their lifespan and causes skeletal muscle atrophy, the autophagic-lysosomal pathway was activated in muscles of the masseter and tongue (Iida et al., 2011). Such activation of the autophagic-lysosomal pathway was associated with significantly lower levels of phosphorylation of signaling effectors that act downstream of mTOR, such as 4E-BP1 and p70 S6K (Iida et al., 2011). Interestingly, similar activation was not detected in the gastrocnemius (Iida et al., 2011). Those researchers speculated that the masseter and tongue move more actively than limb muscles, and that amino acid deficiency in those more active tissues downregulates the mTOR signalling pathway, stimulating the autophagic-lysosomal pathway. These findings suggest that Klotho may regulate autophagy differently in different types of muscle, which future studies should explore.

Further study is even more necessary in light of reports that under some circumstances, Klotho has no significant effect on muscle tissue. For example, recombinant Klotho protein failed to directly influence the proliferation or differentiation of C_2_C_12_ myoblasts in culture (Avin et al., 2018). A cross-sectional study of hemodialysis patients showed that plasma concentration of soluble (free) Klotho did not significantly correlated with these muscle mass (Fukasawa et al., 2014). Thus, much remains to be clarified about the role of Klotho in muscle function and whether autophagy regulation is involved.

#### Klotho and Autophagy in Diabetes

Autophagy plays a key role in diabetes and its complications (Bhattacharya et al., 2018). Both LC3 and Klotho are underexpressed in pancreatic islet β-cells of diabetic patients and in a mouse model of diabetes (*db/db* mice), and such downregulation of Klotho is associated with a decrease in insulin storage in pancreatic β-cells (Lin and Sun 2015). Restoring full-length Klotho expression in *db/db* mice attenuated the development of diabetes, enhanced glucose tolerance, and restored LC3 expression in islet β-cells (Lin and Sun 2015). These findings identify Klotho and autophagy as therapeutic targets in type 2 diabetes mellitus.

Researchers have attributed Klotho’s ability to activate autophagy partially to its antioxidant property, although oxidative stress has also been reported to activate autophagy (Lin and Kuang 2014; Filomeni et al., 2015). However, since autophagy activity cannot be assessed solely based on LC3 expression, more studies are needed to verify the ability of Klotho to regulate autophagy in islet cells as well as to clarify the mechanisms involved.

## Conclusion

**FIGURE 1:**
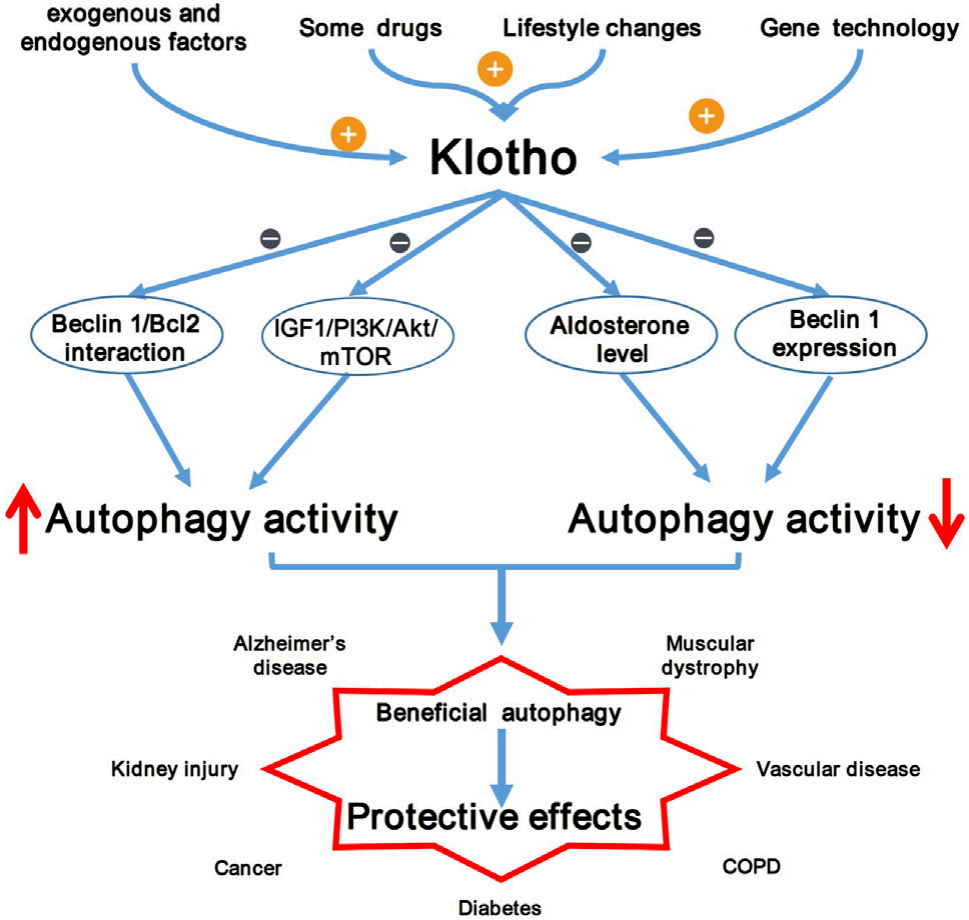
Klotho as potential autophagy regulator and therapeutic target. The expression of Klotho can be upregulated by many exogenous and endogenous factors, some drugs, lifestyle changes and gene technology. Klotho plays a dual role in regulating autophagy (induction or inhibition) through its influence on the IGF-1/PI3K/AKT/mTOR signaling pathway, Beclin 1 expression and activity, and aldosterone level. Klotho exhibits protective effects in many diseases, making it a potential therapeutic target.

Normal autophagy is crucial for homeostasis. When autophagy is insufficient or excessive, it can lead to the occurrence and development of disease. Therefore, restoring normal autophagy is a potential treatment for several diseases. An increasing number of reports have shown that the components required to induce autophagy depend on the nature of the induction signal and the type of cell, and they do not always involve canonical members of the autophagy signaling pathway (Corona Velazquez and Jackson 2018). This may be the reason why Klotho influences autophagy in different ways depending on the tissue and the physiological or pathological conditions. Although its regulatory effects may differ with the situation, Klotho always seems to serve to restore normal autophagy activity, and it therefore shows potential to treat various disorders (Figure 1). This potential has been demonstrated in preclinical studies and some studies of clinical samples involving neurodegenerative disease, kidney disease, cancer, lung disease, vascular diseases and diabetes.

On the other hand, most studies of Klotho and autophagy have assessed the latter by measuring LC3, p62, Beclin1 as well as the number of autophagosomes and autophagolysosomes at a steady state. In fact, some studies have assessed autophagy based on only one or two markers. These approaches give an incomplete picture, since autophagy, a dynamic cellular process, involves several steps: initiation, phagophore, expansion, autophagosome maturation, fusion with the lysosome, cargo degradation in the lysosome and efflux (Kuma et al., 2017). Therefore, it is important to assess autophagic activity at each step or to monitor autophagy flux, which bring us a more objective and comprehensive understanding of the role of Klotho in regulating autophagy.

Future work should further develop the potential of Klotho in prevention and treatment of diseases. It should also explore the effects of Klotho throughout the complete pathway of autophagy and elucidate the molecular mechanisms involved. Studies should investigate whether Klotho-regulated autophagy also plays a role in other aging-related diseases such as osteoporosis, atherosclerosis, heart disease and stroke.
